# Comparison of COVID-19 outcomes in organ transplant recipients (OTr) and non-transplant patients: a study protocol for rapid review

**DOI:** 10.1186/s13643-021-01854-8

**Published:** 2021-11-21

**Authors:** Alexis H. Lerner, Elizabeth J. Klein, Anna Hardesty, Orestis A. Panagiotou, Chelsea Misquith, Dimitrios Farmakiotis

**Affiliations:** 1 The Warren Alpert Medical School of Brown University, 222 Richmond Street, Providence, RI 02906 USA; 2 Department of Medicine, The Warren Alpert Medical School of Brown University, 593 Eddy Street, Providence, RI 02903 USA; 3 Brown University School of Public Health, 121 South Main Street, RI 02906 Providence, USA; 4 Brown University Library, 10 Prospect Street, RI 02912 Providence, USA; 5 Division of Infectious Diseases, The Warren Alpert Medical School of Brown University, 593 Eddy Street, Gerry House 111, Providence, RI 02903 USA

**Keywords:** COVID-19, Organ transplant, Immunosuppression

## Abstract

**Background:**

The COVID-19 pandemic has devastated the global community with nearly 4.9 million deaths as of October 2021. While organ transplant (OT) recipients (OTr) may be at increased risk for severe COVID-19 due to their chronic immunocompromised state, outcomes for OTr with COVID-19 remain disputed in the literature. This review will examine whether OTr with COVID-19 are at higher risk for severe illness and death than non-immunocompromised individuals.

**Methods:**

MEDLINE (via Ovid and PubMed) and EMBASE (via Embase.com) will be searched from December 2019 to October 2021 for observational studies (including cohort and case-control) that compare COVID-19 clinical outcomes in OTr to those in individuals without history of OT. The primary outcome of interest will be mortality as defined in each study, with possible further analyses of in-hospital mortality, 28 or 30-day mortality, and all-cause mortality versus mortality attributable to COVID-19. The secondary outcome of interest will be the severity of COVID-19 disease, most frequently defined as requiring intensive care unit admission or mechanical ventilation. Two reviewers will independently screen all abstracts and full-text articles. Potential conflicts will be resolved by a third reviewer and potentially discussion among all investigators. Methodological quality will be appraised using the Newcastle-Ottawa Scale. If data permit, we will perform random-effects meta-analysis with the Sidik-Jonkman estimator and the Hartung-Knapp adjustment for confidence intervals to estimate a summary measure of association between histories of transplant with each outcome. Potential sources of heterogeneity will be explored using meta-regression. Additional analyses will be conducted to explore the potential sources of heterogeneity (e.g., subgroup analysis) considering least minimal adjustment for confounders.

**Discussion:**

This rapid review will assess the available evidence on whether OTr diagnosed with COVID-19 are at higher risk for severe illness and death compared to non-immunocompromised individuals. Such knowledge is clinically relevant and may impact risk stratification, allocation of organs and healthcare resources, and organ transplantation protocols during this, and future, pandemics.

**Systematic review registration:**

Open Science Framework (OSF) registration DOI: 10.17605/osf.io/4n9d7.

**Supplementary Information:**

The online version contains supplementary material available at 10.1186/s13643-021-01854-8.

## Background

The first cases of infection from severe acute respiratory syndrome coronavirus 2 (SARS-CoV-2), the virus that causes coronavirus disease 2019 (COVID-19), were identified in Wuhan, China, in December 2019 [1]. Since, the COVID-19 pandemic has devastated the global community with nearly 4.9 million deaths as of October 2021 [2]. Organ transplant recipients (OTr) may be at increased risk for severe COVID-19 due to their chronic immunocompromised state. Immunosuppressants may dull the antiviral response, predisposing the host to worse outcomes, and attenuate signs and symptoms of infection, compromising timely diagnosis and management. However, these harms may be counterbalanced by the beneficial dampening effect immunosuppressants have on the cytokine storm and hyperinflammatory state associated with COVID-19.

Empiric studies comparing outcomes of OTr with COVID-19 to those of the general (i.e., non-transplant) patient population have yielded mixed results. Early reports, such as a matched case-control study by Caillard et al. (2020), reported a substantially higher risk of death in hospitalized OTr with COVID-19 (17.9% vs 11.4%, respectively, *p* = 0.038) [3]. The significance of this finding persisted after adjusting for age, body-mass index (BMI), and major comorbidities, suggesting that transplantation status may be an independent risk factor for 30-day mortality in patients with COVID-19. In contrast, other reports have found no significant differences in length of stay (LOS) or clinical outcomes between OTr and non-transplant patients with COVID-19, and question whether chronic immunosuppression impacts COVID-19 outcomes in OTr. For instance, Sharma et al. (2021) found no significant difference in severe disease between OTr and controls; furthermore, OTr were able to mount a higher inflammatory response than controls, and OTr presented with a similar degree of lymphopenia as controls [4]. Interestingly, emerging data suggest mortality may be lower in OTr who are maintained on their chronic immunosuppressive therapy throughout the course of COVID-19 illness. A systematic review and meta-analysis of 202 OTr with COVID-19 found that continuation of an immunosuppressive regimen resulted in lower levels of severe and critical disease [5].

This rapid review will examine whether OTr with COVID-19 are at higher risk for severe illness and death than non-immunocompromised individuals. Compared to that of a traditional systematic review, the methodology of a rapid review is well-suited to summarize emergent evidence for patients, clinicians, and policymakers in a resource-efficient, succinct manner [6]. This information is of high priority during the COVID-19 pandemic: given the clinical vulnerability and uncertainty of management of immunocompromised patients, clinicians and policy makers would benefit from fast access to synthesized information.

## Methods

### Design

This rapid review will compare clinical outcomes of COVID-19 between OTr and patients without a history of transplant (controls). The review protocol has been registered within the Open Science Framework database (osf.io/4n9d7) and is being reported in accordance with the reporting guidance provided in the Preferred Reporting Items for Systematic Reviews and Meta-Analyses Protocols (PRISMA-P) statement [7] (Additional file 1). The rapid review will be conducted following the methodological guidance from the Cochrane Handbook [8].

### Eligibility criteria

- *Population:* Patients 18 years of age and older diagnosed with COVID-19
- *Exposure:* History of solid organ transplant (kidney, liver, heart, lung, pancreas, and intestine, excluding bone marrow or hematopoietic stem cell)
- *Comparison:* Patients with no history of transplant (studies must contain a control group to be included)
- *Outcomes:*
  -*Primary:* mortality as defined in each study, with possible further analyses of in-hospital mortality, 28 or 30-day mortality, and all-cause mortality versus that which is attributable to COVID-19;
  -*Secondary:* severity of COVID-19 disease, most frequently defined as requiring intensive care unit admission or mechanical ventilation
- *Study design:* Observational studies (cohort and case-control design)
- *Timing:* Published between December 1, 2019, and October 31, 2021
- *Setting:* No restriction
- *Language:* Written in English

### Information sources and search strategy

Database searches will be conducted in MEDLINE (via Ovid and PubMed) and EMBASE (via Embase.com) for articles published between December 1, 2019, and October 31, 2021. The search strategy will be peer-reviewed by a research librarian in accordance with the Peer Review of Electronic Search Strategies (PRESS) 2015 Guideline Statement [9]. The Cochrane Library will not be searched as it focuses on effectiveness of interventions, which is not the focus of this research question.

The search will include a broad range of terms and keywords related to COVID-19 and organ transplant. COVID-19 concept search terms will be based on a previously designed search strategy on COVID-19 and telemedicine designed at Mayo Clinic Libraries on April 6, 2020 [10]. Organ transplant concept terms will be modified from the Ovid MEDLINE search strategy proposed by Raja et al. [11]. A draft search strategy for MEDLINE is provided in Additional File 2.

### Screening and selection procedure

All articles identified from the literature search will be independently screened by two reviewers. First, titles and abstracts of articles returned from initial searches will be screened based on the eligibility criteria outlined above. Potential conflicts will be resolved by a third reviewer and potential discussion among all investigators. Second, full texts will be examined in detail and screened for eligibility. A flow chart showing the numbers of studies included and excluded at each stage of the study selection process will be provided. Reasons for exclusions will be provided for studies screened at the full-text level.

### Data extraction

Two reviewers will extract data from each study independently using Covidence as an online data extraction platform. Data will be extracted from the original reports into specially designed and pilot-tested data extraction forms designed to serve the investigation of the primary and secondary outcomes described above. Conflicts will be resolved by a third reviewer and potentially discussion among all investigators. Given the time-sensitive nature of this rapid review, original data from the study investigators will not be sought for confirmation. Reviewers will enter data in electronic extraction forms. The following data will be extracted: first author, year of publication**,** country and continent of publication, volume of transplant center, transplant and comparator population demographics (i.e., age, race, ethnicity, and sex), transplant-specific characteristics (organ type and respective number of transplants), comparator-specific characteristics (matching criteria, waitlist status, chronic kidney disease [CKD], end-stage renal disease [ESRD], and dialysis), and period (quintile: Q) of data collection (Q1: March-June 2020; Q2: July-October 2020; Q3: November 2020-February 2021; Q4: March-June 2021; Q5: July-October 2021). Given that the first case of COVID-19 in the USA was reported in January 2020, we expect very few, if any, studies with data collected prior to March 2020; if such studies are identified, they will be included in Q1.

The risk of bias/quality assessment of primary studies will be evaluated using the Newcastle-Ottawa Scale (NOS) for observational (e.g., cohort and case-control) studies [12]. Using the NOS tool, each study is judged on eight items categorized into three groups: the selection of the study groups, the comparability of the groups, and the ascertainment of either the exposure or outcome of interest for case-control or cohort studies, respectively. Stars are awarded for each quality item, and the highest quality studies are awarded up to nine stars. We will consider studies with 0-3, 4-6, and 7-9 stars to represent low, moderate, and high-quality studies, respectively. The risk of bias for each study will be independently assessed by two reviewers. Discrepant scores will be resolved by a third reviewer and potentially discussion among all investigators.

### Data synthesis

We will perform narrative synthesis using evidence tables and evidence maps to describe the PICOTS elements, including study characteristics (e.g., author, journal, study design), population characteristics (e.g., eligibility, type of transplant, age distribution, demographics, definitions of COVID positivity), outcome operationalization (e.g., definitions of mortality, measures of effect estimates), covariates for which statistical models are adjusted, and findings. We will also assess whether covariates in adjusted models overlap sufficiently so that the reported conditional effects are estimates of the same estimand. Based on these elements, we will assess clinical and methodological heterogeneity and whether studies are statistically exchangeable to be meta-analyzed. The decision to meta-analyze is based on assumptions that are not testable with observed data as acknowledged by the Cochrane Collaboration [8]. Hence, we will quantitatively synthesize findings across studies through meta-analysis if our evidence tables and maps demonstrate that studies are similar enough in their design, the populations have similar characteristics, the two comparison groups (transplant vs non-transplant patients) are defined in the same way across studies, outcomes are operationalized in a consistent way in the body of evidence, and the reported effects are conditional estimates of the same estimand.

If the assumptions of study exchangeability are reasonably met, we will perform random-effects meta-analysis with the Sidik-Jonkman estimator and the Hartung-Knapp adjustment for confidence intervals to estimate a summary measure of association between history of transplant and each outcome. We are interested in binary outcomes for which we expect that studies report the measures of odds ratio, risk ratio, risk difference, or hazard ratio. To quantify statistical heterogeneity, we will use the *I*^2^ statistic: the percentage of variance among studies that is attributable to genuine underlying differences in study properties rather than measurement error. We will explore potential sources of heterogeneity (e.g., organ type, number of years post-transplant, number of transplants, transplant center volume, US versus non-US studies, period of data collection) using random effects meta-regression. These parameters are selected as they can influence outcomes through host factors (organ type, number of years post-transplant, number of transplants) or different practices (including available treatments) in the management of COVID-19 (US versus non-US studies, period of data collection). We will assess the presence of small-study effects visually with funnel plots and statistically using Egger’s regression; both methods provide evidence for whether smaller vs larger studies give systematically different results, which may be due to publication bias as well as other meta-biases [13].

Although both adjusted and unadjusted estimates of association will be extracted, we will synthesize separately those for which primary studies have performed some adjustment for confounders, e.g., through regression, matching, or other approach.

The Newcastle-Ottawa Scale (NOS) will be used to evaluate study quality as it is validated, adaptable, and useful as a potential moderator in systematic reviews and meta-analyses.

### Additional analyses

Given the changing temporal and geographic landscape of the COVID-19 pandemic through the study inclusion period, we will perform subgroup analyses accounting for period of data collection, country and continent of study, and transplant center volume. We will also perform subgroup analyses for type of transplanted organ. To assess the role of methodological quality, we will perform subgroup analyses according to study quality determined by the NOS. We expect that studies will already report associations adjusted for covariates, which we will identify according to the publications. We will not adjust for confounders that are not included in the primary studies. If there is substantial variability in the covariates, we will consider potential categorizations (e.g., demographics, clinical characteristics, COVID-19 prevalence) and perform analyses according to the relevant subgroup, if feasible. Finally, we will perform random-effects meta-regression to explore factors that may drive statistical heterogeneity.

### Confidence in cumulative evidence

To assess the trustworthiness of the body of evidence and our confidence in the synthesized findings, we will use six quantitative criteria previously proposed [14] including level of statistical significance, sample size, statistical significance for the largest study, 95% prediction intervals, between-study heterogeneity, and the results of tests for small study effects.

### Software considerations

All analyses will be conducted in Stata version 17 (StataCorp LP, College Station, TX, USA) and R (R Foundation, Vienna, Austria).

## Discussion

Although descriptive reviews of SARS-CoV-2 infection in transplant recipients have been published [3, 4, 15], we expect this to be an informative meta-analysis of studies comparing COVID-19 outcomes in transplant and non-transplant patients. Such clinically relevant knowledge may impact risk stratification, allocation of organs and healthcare resources, and organ transplantation protocols during this, and future, pandemics.

At the rapid review level, population and reporting differences between studies may limit the generalizability of our results. Variation in patient demographic reporting, outcome reporting (e.g., in-hospital mortality vs 28 or 30-day, all-cause vs attributable mortality), and matching parameters may render it difficult to form cohesive inferences. Furthermore, the COVID-19 pandemic has propagated in waves across time and geography. Since this review will include studies published over an extended period without geographic restriction, each study cohort may have experienced a vastly different infection rate, which could have resulted in variable outcomes. To address study-level limitations, we will conduct temporal and geographic sub-analyses accounting for period of data collection, country and continent of study, and transplant center volume.

Any amendments made to this protocol when conducting the review will be outlined in OSF and reported in the final manuscript.

Results will be disseminated through conference presentations and publication in a peer-reviewed journal. Through these routes, we expect this rapid review comparing outcomes of COVID-19 between OTr and non-transplant patients to inform best practice among clinicians, hospitals, researchers, and policymakers as they address the needs of the OTr population during the ongoing and future pandemics.

## Supplementary Information

**Additional file 1.** PRISMA-P (Preferred Reporting Items for Systematic review and Meta-Analysis Protocols) 2015 checklist: recommended items to address in a systematic review protocol*

**Additional file 2.** Search Strategy

## Data Availability

Data and other pieces of information are available at 10.17605/osf.io/4n9d7.

## Abbreviations

SARS-CoV-2: Severe acute respiratory syndrome coronavirus 2
COVID-19: Coronavirus disease 2019
NOS: Newcastle-Ottawa Scale
OT: Organ transplant
OTr: Organ transplant recipients
OSF: Open Science Framework (OSF)
CKD: Chronic kidney disease
ESRD: End-stage renal disease
