# Supplementary material for: Comparison of COVID-19 outcomes in organ transplant recipients (OTr) and non-transplant patients: a study protocol for rapid review
Source: Syst Rev. 2021 Nov 21;10:299. doi: 10.1186/s13643-021-01854-8 (PMC8606222; doi:10.1186/s13643-021-01854-8)
Supplement: Supplementary file 2 — Additional file 2. Search Strategy [file 13643_2021_1854_MOESM2_ESM.doc]

Database: Ovid MEDLINE(R) and Epub Ahead of Print, In-Process, In-Data-Review & Other Non-Indexed Citations and Daily <1946 to March 16, 2021>

Date Run: March 17, 2021

Search Strategy:

--------------------------------------------------------------------------------

1 exp COVID-19 Vaccines/ or exp COVID-19/ or exp COVID-19 Testing/ (65240)

2 SARS-CoV-2/ (50694)

3 ((("Corona virinae" or "corona virus" or Coronavirinae or coronavirus or COVID or nCoV) adj4 ("19" or "2019" or novel or new)) or (("Corona virinae" or "corona virus" or Coronavirinae or coronavirus or COVID or nCoV) and (wuhan or china or chinese)) or "Corona virinae19" or "Corona virinae2019" or "corona virus19" or "corona virus2019" or Coronavirinae19 or Coronavirinae2019 or coronavirus19 or coronavirus2019 or COVID19 or COVID2019 or nCOV19 or nCOV2019 or "SARS Corona virus 2" or "SARS Coronavirus 2" or "SARS-COV-2" or "Severe Acute Respiratory Syndrome Corona virus 2" or "Severe Acute Respiratory Syndrome Coronavirus 2").ti,ab,hw,kw. (114450)

4 1 or 2 or 3 (114450)

5 exp Transplants/ (25984)

6 exp Organ Transplantation/ (214918)

7 transplantation/ or Tissue Transplantation/ or cold ischemia/ or transplantation, autologous/ or transplantation, heterologous/ or transplantation, heterotopic/ or exp transplantation, homologous/ (174616)

8 exp Transplant Recipients/ (4497)

9 tissue donors/ or living donors/ or unrelated donors/ (54802)

10 transplant*.ti,ab,kw,jw. (539854)

11 graft*.kf,ab,ti. (345371)

12 (allograft* or allo-graft*).kf,ab,ti. (69465)

13 (allotransplant* or allo-transplant*).kf,ab,ti. (5135)

14 (autograft* or auto-graft*).kf,ab,ti. (17639)

15 (autotransplant* or auto-transplant*).kf,ab,ti. (7551)

16 (homograft* or homo-graft*).kf,ab,ti. (6313)

17 (homotransplant* or homo-transplant*).ab,ti,kf. (1572)

18 (isograft* or iso-graft*).ab,ti,kf. (1992)

19 (isotransplant* or iso-transplant*).ab,ti,kf. (281)

20 (posttransplant* or post-transplant*).ab,ti,kf. (42993)

21 (pretransplant* or pre-transplant*).ab,ti,kf. (13574)

22 (re-transplant* or retransplant*).ab,ti,kf. (5745)

23 (organ adj2 (donor* or recipient*)).ab,ti. (13250)

24 or/5-23 (882854)

25 4 and 24 (1602)

26 exp Animals/ (23900993)

27 exp Humans/ (19100346)

28 26 not 27 (4800647)

29 25 not 28 (1602)

30 ("32654332" or "32890139" or "33350626" or "33151337" or "33573820" or "33478747" or "33443778" or "32779808" or "33278850" or "33196624" or "33284498" or "33406279" or "32844546" or "33363263" or "32780493" or "33043597" or "32866433" or "33252201").ui. (18)

31 29 and 30 (18)

32 limit 29 to english language (1566)

***************************

Line 3: modified from the [COVID-19 and Telemedicine](https://www.mlanet.org/page/covid-19-literature-searching) search by Ellen Aaronson at Mayo Clinic Libraries on April 06, 2020.

Lines 10-23: modified from the following article (Supplementary Material 2 contains the search strategy):

Raja MA, Mendoza MA, Villavicencio A, Anjan S, Reynolds JM, Kittipibul V, Fernandez A, Guerra G, Camargo JF, Simkins J, Morris MI, Abbo LA, Natori Y. COVID-19 in solid organ transplant recipients: A systematic review and meta-analysis of current literature. Transplant Rev (Orlando). 2021 Jan;35(1):100588. <https://doi.org/10.1016/j.trre.2020.100588>
